# Bone loss and new vertebral fractures during treatment with romosozumab: a case-report

**DOI:** 10.1007/s11657-024-01367-6

**Published:** 2024-01-19

**Authors:** Anneke F. Marsman, Renate T. de Jongh, Bernd P. Teunissen, Willem F. Lems

**Affiliations:** 1 Department of Rheumatology, Amsterdam University Medical Center, VUmc, De Boelelaan 1117, 1081 HV Amsterdam, the Netherlands; 2 Department of Endocrinology and Metabolism, Amsterdam University Medical Center, VUmc, Amsterdam, the Netherlands; 3 Amsterdam Movement Sciences, Aging & Vitality and Muskuloskeletal Health, Amsterdam, The Netherlands; 4 Department of Radiology and Nuclear Medicine, Amsterdam University Medical Center, VUmc, Amsterdam, the Netherlands; 5 Department of Rheumatology, Amsterdam Rheumatology and Immunology Center, Reade, Amsterdam, The Netherlands

**Keywords:** Osteoporosis, Romosozumab, Vertebral fractures

## Abstract

**Purpose:**

This study aimed to illustrate the possibility of an unfavorable response to treatment with the anabolic agent romosozumab for patients with severe osteoporosis and to discuss explanations for treatment failure.

**Methods:**

Dual-energy x-ray absorptiometry (DXA) including vertebral fracture assessment (VFA) and X-rays of the thoracolumbar spine was used to assess bone mineral density (BMD) and the presence of vertebral fractures before and after treatment with romosozumab.

**Results:**

Our patient developed a decrease in the BMD of the hip, two incident new vertebral fractures, and worsening of one prevalent vertebral fracture during 1 year treatment with romosozumab. We have not detected non-adherence, there was no pretreatment with anti-resorptives, and we observed no signs of secondary osteoporosis and/or comorbidities.

**Conclusion:**

As the number of patients treated with romosozumab is rising, it becomes more likely that more patients will be found with new fractures and/or an unfavorable BMD response. Probably, the unfavorable response is a (bad) chance finding, but we think it is crucial for clinicians and patients to exclude nonadherence, new comorbidities and pretreatment with anti-resorptives as explanation in these patients.

An 83-year-old woman presented at the fracture liaison service of our outpatient clinic 3 months after a fracture of the distal radius following a fall from standing height. Her wrist fracture healed with conservative treatment. History revealed chronic back pain and a period of weight loss for which she had visited an internal medicine specialist, who did not find an explanation for that, based on, amongst other data, a CT-scan of the abdomen.

At additional questioning she reported her menarche at 15 years of age and her menopause around her 50th birthday. Dietary calcium intake was adequate and she used to exercise at a regular basis (tennis, walking, cycling) for at least 30 min a day. She never smoked, did not use alcohol, and her family history mentioned a first degree relative with a hip fracture (father, at 80 years of age who was diagnosed with bone cancer).

Her medical history included dyslipidemia and a vitamin B12 deficiency. She used omeprazole, rosuvastatin, and temporarily buprenorphine (transdermal) and oxazepam for her back pain.

At physical examination, her height was 159 cm (prior 168 cm), weight 55 kg (body mass index 21.8 kg/m^2^), and examination of her back showed a severe thoracolumbar scoliosis and thoracic kyphosis without local vertebral (pressure) pain. Examination of her heart, lungs, abdomen, and lymph nodes was normal.

A dual-energy x-ray absorptiometry (DXA) including vertebral fracture assessment (VFA) and X-rays of the thoracolumbar spine (due to difficult interpretation of VFA alone, as a result of the severe scoliosis and osteopenia) was conducted. They showed osteoporosis of the lumbar spine and total hip (*T*-scores, respectively, − 3.2 and − 3.1) (Table 1) and vertebral fractures (Th12 Genant grade 3, L1 Genant grade 1; Fig. 1) without signs of pathologic fractures. *T*-scores of fracture-free vertebrae L2, L3, and L4 were respectively − 3.8, − 4.1, and − 3.0.

**Table 1:** DXA and bone marker results before, during, and after treatment with romosozumab

	16–09-21 Prior to romosozumab	13–01-22 At 2 months	11–10-22 At 12 months	12–01-23 2 months after the last injections romosozumab and additional treatment with zoledronic acid
CTx (ng/L) *Normal range 0–1008 ng/L*	813	674	589	76
P1NP (ug/L) *Normal range 16–96 ug/L*	89	279 (↑)	65	21
Lumbar spine				
* T*-score	− 3.2		− 3.1	
BMD (g/cm^2^)	0.693		0.708	
BMC (g)	50.43		39.71	
Area (cm^2^)	72.78		56.08	
Total hip				
* T*-score	− 3.1		− 3.5	
BMD (g/cm^2^)	0,563		0.516	
BMC (g)	22.15		20.58	
Area (cm^2^)	39.33		39.85	
Femoral neck				
* T*-score	− 3.3		− 3.5	
BMD (g/cm^2^)	0.484		0.461	
BMC (g)	2.51		2.51	
Area (cm^2^)	5.19		5.43	

*CTx* carboxy-terminal collagen crosslinks, *P1NP* procollagen type I N-terminal propeptide, *BMD* bone mineral density, *BMC* bone mineral content

**Fig. 1:**
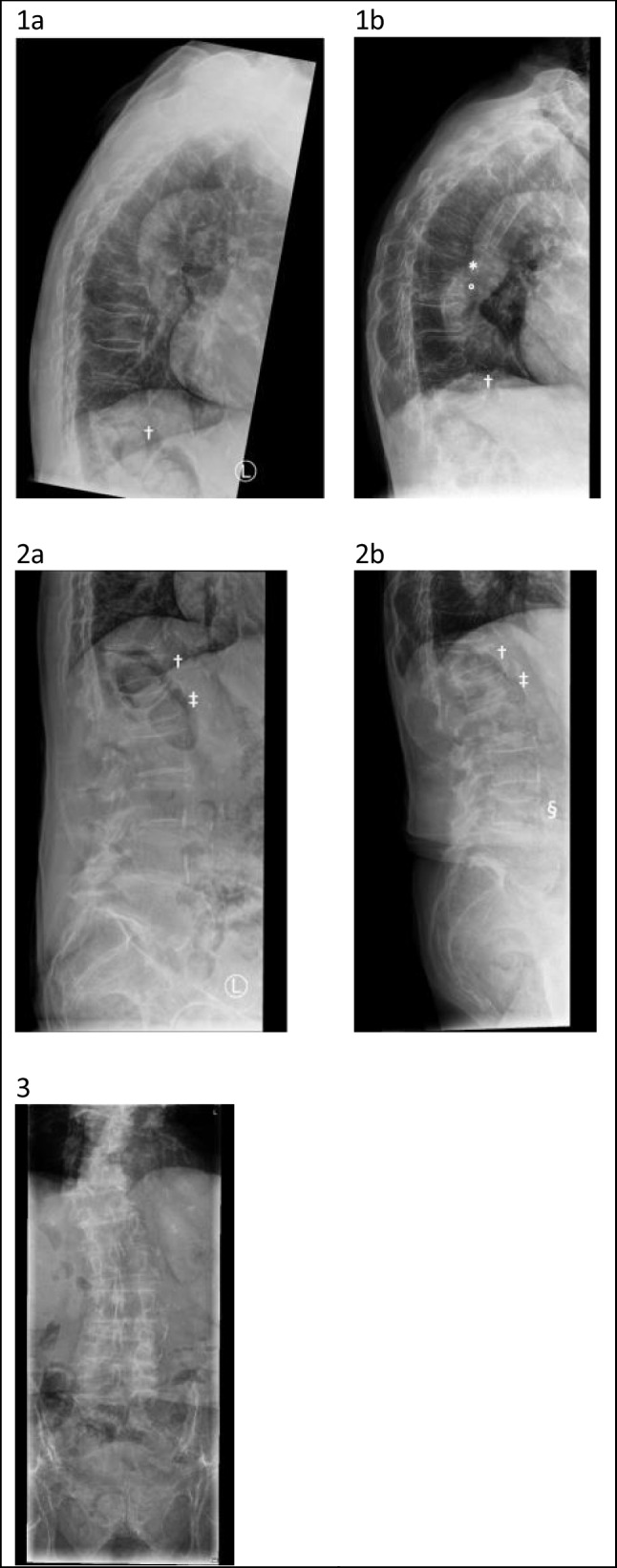
X-rays of the thoracolumbar before and after treatment with romosozumab. Thoracic spine before (1a) and after (1b) treatment with romosozumab. Lumbar spine before (2a) and after (2b) treatment with romosozumab. Thoracolumbar scoliosis (3). * = fracture Th8. ° = fracture Th9. † = fracture Th12. ‡ = fracture L1. § = Fracture L4

Blood tests showed an erythrocyte sedimentation rate (ESR) of 17 mm/h (normal < 30), no monoclonal protein, normal blood count, kidney function, liver enzymes, alkaline phosphatase, HbA1c, calcium, phosphate, (para)thyroid hormone concentrations, celiac screen, 25-hydroxyvitamin-D, and B12. Carboxy-terminal collagen crosslinks (CTX, a marker for bone resorption) and procollagen type I N-terminal propeptide (P1NP, a marker for bone formation) were in the normal range for postmenopausal women (Table 1). Urinary calcium excretion was normal. Thus, no underlying secondary causes were identified.

We concluded a high fracture risk in an 83-year-old lady with low bone mineral density (BMD) in the osteoporotic range and multiple fractures including vertebral fractures. Risk factors for the low BMD were her low body weight and her positive family history. Also, the use of proton pomp inhibitors might be related to lower BMD [1]. Further laboratory examination did not reveal secondary causes of the low BMD. Her 10-year probability for a major osteoporotic fracture was 45% and 38% for a hip fracture, calculated using Fracture Risk Assessment Tool (FRAX). Because of the very high fracture risk, the preferred treatment was an anabolic drug and she started treatment with romosozumab subcutaneous injections for 1 year [2].

During treatment with romosozumab, our patient did not experience any physical trauma, nor an episode of new backpain or other symptoms related to a possible secondary cause of her osteoporosis. Her height and weight remained stable. During evaluation after 1 year of treatment, our patient confirmed that she had taken her monthly (two) injections.

We evaluated the spine and BMD using both DXA, VFA and X-rays of the thoracolumbar spine. Unexpectedly, new vertebral fractures were present of the thoracic and lumbar spine (Th8 Genant grade 2, Th9 Genant grade 3, L4 Genant grade 1), increased collapse of fracture L1 (now Genant grade 2), and a stable fracture of Th12 (Genant grade 3) (Fig. 1). Moreover, we found a decrease in BMD of both the total hip (8.4%) and the femoral neck (4.8%) and a minimal increase of the BMD of the spine (2.2%) (Table 1). The imaging of the two lumbar spine measurements is more difficult to interpret, due to the severe thoracolumbar scoliosis of the patient, which might also have interfered with the measured bone area.

Repeated blood tests again showed no signs of a secondary cause, with an ESR of 16 mm/h (normal < 30), no monoclonal protein, normal blood count, kidney function, liver enzymes, alkaline phosphatase, calcium, phosphate, and (para)thyroid hormone concentrations.

Following completion of treatment with romosozumab, our patient was administered zoledronic acid in November 2022.

## Discussion

We presented an 83-year-old women with a very high fracture risk who demonstrated a decrease in the BMD of the hip, incident new vertebral fractures, and worsening of prevalent vertebral fractures during romosozumab treatment.

This was highly unexpected based on previous results of romosozumab in RCTs. These demonstrate, on a group level, a large mean increase in BMD of the spine and of the hip, and a reduction in incidence of vertebral fractures, compared to placebo and alendronate as active comparators [3, 4].

The most important question is why did this patient not show the expected response to romosozumab regarding BMD and fracture prevention? There are several potential explanations:

1. Non adherence to therapy. Although lack of adherence is quite common in the field of osteoporosis, not only with bisphosphonates but also with parenteral drugs [5], we have two arguments against this:
  - patient confirms that she has used all her medication over 12 months and delivery of romosozumab was confirmed by the pharmacy. In The Netherlands, romosozumab is supplied by a central pharmacy (Apotheek Zorg) who is also responsible for patient instruction (done by trained nurses) and follow-up phone calls including motivational interviews every 3 months, to assess side-effects and adherence. Earlier, this has been shown to increase the two-year persistence in patients treated with teriparatide [6].
  - the pattern of changes in bone markers, with an early rise after 2 months in P1NP and a decrease over time in CTX, reflect the changes observed in RCTs with romosozumab [3, 4].
2. Another illness or comorbidity during the 1-year treatment with romosozumab. We have seen the patient again after 12 months treatment with romosozumab at our outpatient clinic. Nor history nor physical examination nor the repeated laboratory tests revealed a new cause of secondary osteoporosis.
3. Pretreatment with anti-resorptive osteoporotic drugs: it is well known that the effects of anabolic drugs, including romosozumab, can be blunted after pretreatment with anti-resorptives [7, 8]. However, this patient was not pre-treated with any antiresorptive drug.
4. Misfortune, due to the combination of the high-risk for fracture patient profile and the fact that to date no anti-osteoporotic drug can provide 100% fracture reduction. In the ARCH and FRAME trial data at 12 months, the mean increase in BMD at the lumbar spine was 13.7% and 13.1%, respectively, and total hip 6.2% and 6.0% [3, 4]. However, in 1.1% of patients, romosozumab treatment did not result in an increase in BMD and 0.5–4% experienced a new vertebral fracture as our patient, with the highest percentage in high-risk for fracture patients in the ARCH trail [3, 4]. Of this patient subgroup, only half received fewer than six doses of romosozumab [4]. Real-world data from two Japanese cohort studies reported 3% and 5.7% of patients who suffered new fractures during treatment with romosozumab, of which 1.3% and 1.9% a vertebral fracture [9, 10]. A recent retrospective study regarding 92 romosozumab non-responder patients found an absent early rise in P1NP to be a predictor of nonresponse at month 12 [11]. Our patient however did show an early threefold rise in P1NP.

In our case, the first three explanations did not seem to contribute to the decrease in BMD of the hip and the development and worsening of vertebral fractures during treatment. The fourth explanation cannot be excluded as a contributor. Another point is the scoliosis; the vertebral fractures were difficult to score, but DXA measurement of the hips was reliable and detected unexpected bone loss.

To conclude, based on the data of this patient, non-adherence was unlikely, and we excluded new comorbidities and pretreatment with anti-resorptives. This suggests that our patient had back luck and was one of the small number of patients with new fractures and a decrease in BMD during romosozumab treatment. Since the number of patients treated with romosozumab is rising, due to chance, it becomes more likely that more patients will be found with new fractures and/or an unfavorable BMD response. However, it should be highlighted that it is crucial to exclude nonadherence, new comorbidities, and pretreatment with anti-resorptives as explanation in these patients.

## Declarations

The corresponding author (AF Marsman) confirms on behalf of all authors that all three criteria of the authorship criteria and contributions were met.
